# Dr. James T. Whittaker

**Published:** 1900-07

**Authors:** 


Dr. James T. Whittaker, of Cincinnati, O., died at his home in that city, June 5, 1900. Dr. Whittaker was one of the most celebrated clinicians of the period, a teacher of renown, an author of distinction, and a practitioner famous throughout the country. His contributions to the literature of medicine were important in character and his death is a distinct loss to the guild. He was graduated in 1866, but, singularly enough, none of the notices published give his age. He was, however, comparatively a young man to have attained his great distinction.

